# Research on Space Targets Simulation Modulation Algorithm Combined Global–Local Multi-Spectral Radiation Features

**DOI:** 10.3390/s25092702

**Published:** 2025-04-24

**Authors:** Yu Zhang, Songzhou Yang, Zhipeng Wei, Jian Zhang, Bin Zhao, Dianwu Ren, Jingrui Sun, Lu Wang, Taiyang Ren, Dongpeng Yang, Guoyu Zhang

**Affiliations:** 1 State Key Laboratory of High Power Semiconductor Lasers, Changchun University of Science and Technology, Changchun 130022, China; 2 School of Opto-Electronic Engineering, Changchun University of Science and Technology, Changchun 130022, China

**Keywords:** global–local radiation features, multi-source information fusion, space target simulation, non-uniformity compensation, modulation algorithm

## Abstract

To solve the international problem of global–local radiation features simulation of multi-spectral space targets, this paper proposes a multi-spectral space target simulation modulation algorithm that can combine global–local spectral radiation features. An overall architecture of a series-parallel multi-source information fusion space target simulation system (MITS) is constructed, and a global–local multi-spectral radiation feature modulation link is built. A multi-spectral feature modulation algorithm consisting of three modules, including optical engine non-uniformity compensation, global spectral radiant energy modulation, and local radiant grayscale modulation, is designed, and an experimental platform is built to verify the correctness and advancement of the proposed algorithm. The results indicate that the non-uniformity is better than 3.78%, the global simulation error is better than −4.56%, and the local simulation error is better than 4.25%. It is one of the few multi-spectral target simulation modulation algorithms worldwide that can combine the global whole and local details. It supports the performance test and technology iteration of multi-spectral optical loads. It helps to supplement the theoretical system of multi-spectral space target simulation and enhance the ground-based semi-physical simulation link of optical loads.

## 1. Introduction

The spectrum, often referred to as opto-genetic, represents the intensity distribution of light signals at different wavelengths and characterizes the intrinsic properties of the target in terms of reflection or transmission of light [1]. Multi-source space detection technology is based on the unique characteristics of the spectrum through the integration of spectral information of different bands to overcome the defects of stray light, darkness, blurring, and other information loss. It detects a broader range of wavelength bands to significantly improve the accuracy and reliability of target recognition [2], to realize “see through the appearance to perceive the essence”, and become the “piercing eye” to perceive the matter. It is the frontier research direction in space situational awareness and target detection [3]. With the improvement of ground semi-physical simulation links for optical loads [4] and the development of technologies such as digital simulation and deduction of major flight events in the aerospace field [5], multi-spectral space target simulation technology [6] that can provide something close to the real space environment (geometric and radiation characteristics) on the ground has become the basic condition guarantee and key technology path for ground performance testing and verification of multi-source space detection technology. In recent years, it has garnered increasing attention and research, particularly in areas such as simulation accuracy [7], spectral range [8], non-uniformity [9], and contrast ratio [10].

In 2013, OPTRA Inc. proposed a dual-band infrared scene projection method with a spectral range covering two atmospheric windows of 3–5 μm and 8–12 μm, and fusion between infrared images was realized by point-by-point pixel fusion to increase the contrast ratio to 400:1. Still, the method could not change the ratio of the radiation in the two bands [11]. In 2016, Inframet Inc. successively developed Simit and Simat multi-band target simulators with output radiation bands of 1.1–8 μm and 0.4–12 μm for primary and secondary targets, respectively. The simulators can achieve simulated target switching by changing targets but cannot achieve target dynamic continuous simulation [12,13]. In 2016, SBIR Inc. developed a two-color target simulation system based on thin-film resistor arrays. This system obtained high grayscale resolutions of 12 bits at 3–5 μm and 8–12 μm, but it suffered from a large alignment error of the pixels at the edges of the device [14]. In 2018, Pan et al. proposed a zoom scene projection method based on two digital micromirror devices (DMDs) covering two bands of 3.7–4.8 μm and 8–12 μm to achieve the simulation of a dynamic scene. However, the contrast of the simulated images was low [15]. In order to solve the above problem of low spectral modulation accuracy in multi-spectral simulation based on composite multi-display devices, in 2023, Liu and Yun et al. successively carried out research on the multi-spectral simulation method based on the composite-band light source [16,17]. They achieved the simulation of the radiative characteristics of single-star point targets with different color temperatures in the visible range of 450–1000 nm and 400–800 nm with spectral scaling and modulation capability, respectively. Modulation capability can currently only be applied to the simulation of point source targets. For this reason, in 2024, Yang et al. proposed an optical simulation method based on the hybrid of UV LEDs and xenon lamps [18], which achieved the simulation of surface targets in the range of 320–760 nm. After that, they further broadened the spectral bands and proposed a series-parallel hybrid multi-source information fusion space target simulation system [19], which achieved the global spectral radiant energy modulation in the spectral bands of 0.32–12 μm.

The above research shows that the focus of multi-spectral space target simulation methods has evolved from the width of spectral bands to the ability to modulate the global spectral radiation ratio between different spectral bands. However, the spectral radiation features of space targets in the same field of view in the same area at different moments may also be distinct [20], with complex and variable global–local radiation features. Whether the existing multi-spectral simulation method based on the composite band light source or the composite multi-spectral display device cannot simulate the local spectral features of multi-spectral space targets has not yet broken through the technical bottleneck of not being able to simulate the global–local radiation features of the same multi-spectral space target. To this end, this paper aims to achieve the global–local spectral features simulation of multi-spectral space targets, construct an MIST architecture, and propose a multi-spectral spatial target simulation method that combines global and local spectral features. Establishing a link consisting of non-uniformity compensation for each spectral band optical engine, global spectral radiant energy modulation, and local radiant greyscale modulation, a global–local space target simulation algorithm is designed to achieve the global–local spectral feature simulation of multi-spectral space targets.

## 2. Multi-Spectral Modulation Framework with Combined Global–Local Radiation Features

### 2.1. Overall Architecture of an MIST

**Figure 1:**
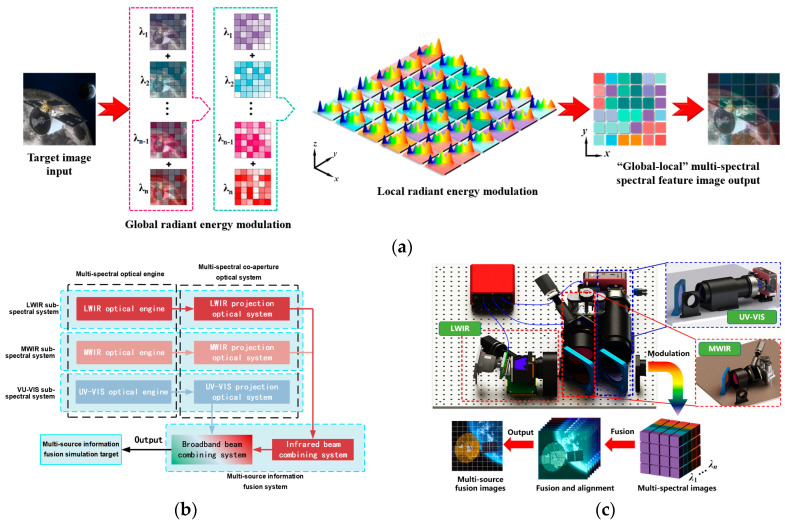
Schematic diagram of MITS. (**a**) Simulation modulation algorithm schematic diagram. (**b**) System composition diagram. (**c**) Working principle diagram.

A space target simulation modulation algorithm with combined global–local multi-spectral radiation features is shown in Figure 1a. In order to combine the differentiated simulation of spectral radiation features in different areas of the same field of view, the target image signal needs to be transferred to different simulation spectral bands. This involves modulating the output energy of each spectral band to achieve global radiant energy modulation, controlling the grayscale of each display device to achieve local radiant energy modulation, and, finally, fusing the modulated image to achieve a common aperture output.

The overall architecture of MITS is divided into a UV–VIS sub-spectral system, an MWIR sub-spectral system, an LWIR sub-spectral system, and a multi-source information fusion system, as shown in Figure 1b. The UV part of MITS and the VIS part of MITS are the first series to form a UV–VIS sub-spectral system and then in parallel with the MWIR and LWIR sub-spectral systems in turn. The UV–VIS, MWIR, and LWIR sub-spectral systems consist of their optical engines (consisting of adjustable illuminating sources, uniform lens, and displays) and projection lens, and the three sub-spectral systems are connected by a multi-source fusion system (consisting of an infrared beam combining system and a broadband beam combining system).

The operating principle of MITS is shown in Figure 1c. The radiation sources and uniform lens of the UV–VIS sub-spectral system, the MWIR sub-spectral system, and the LWIR sub-spectral system provide uniform and controllable illumination beams for the respective display devices. After the display devices modulate the beams, the image modulated by the projection lens at the focal plane of the object is projected and collimated. Parallel light from each spectral band enters the multi-source information fusion system to complete the superposition of beams. Finally, a common aperture composite output covering the parallel beam’s spectral band from UV to LWIR is achieved.

### 2.2. Construction of Multi-Spectral Radiation Features Modulation Link

**Figure 2:**
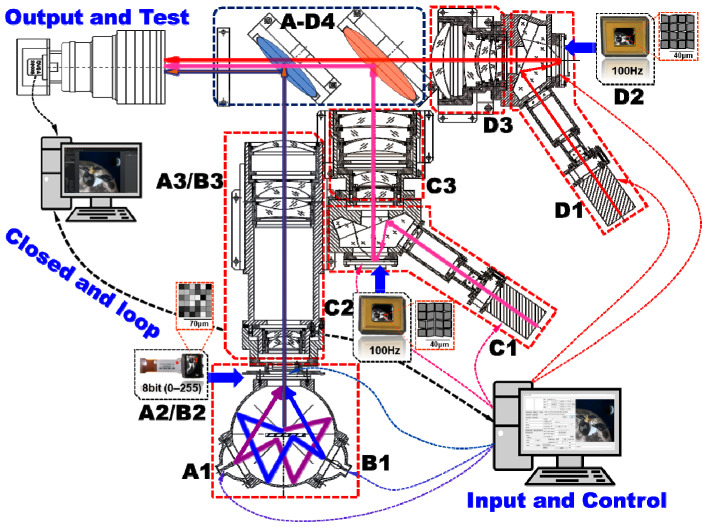
Optical path diagram of the modulation link. (A, B, C, and D represent UV, VIS, MWIR, and LWIR sub-systems. 1, 2, 3, and 4 represent the radiation sources, the display devices, the projection lens, and the multi-source fusion system).

The three steps of the global–local multi-spectral radiant feature modulation link built in this study are the non-uniformity compensation of each spectral optical engine, the global spectral radiant energy ratio modulation, and the local spectral radiant energy ratio modulation [21]. The optical path diagram for the modulation link is shown in Figure 2. MITS adopts the image-side coupling beam form, and each sub-system has a relatively independent radiation source, display device, and projection lens, so it is easier to realize the individual control of each spectral band and multi-spectral radiation energy modulation, finally achieving multi-spectral information fusion.

The steps of the global–local multi-spectral radiation feature modulation link are as follows:

(1) Optical engine non-uniformity compensation

**Figure 3:**
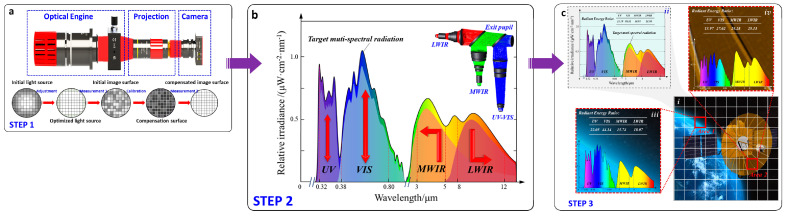
Multi-spectral radiation feature modulation link. (**a**) Schematic diagram of step 1. (**b**) Schematic diagram of step 2. (**c**) Schematic of step 3.

The uniform light from the illumination radiation source enters the test camera through the display device and the projection lens system, and the illumination distribution information of the target surface of the display device is obtained by using position partitioning and grayscale grading; the response function is obtained and fitted to the response curve, and the correction function is calculated to realize the compensation of non-uniformity, as shown in Figure 3a.

(2) Global spectral radiant energy modulation

The required power of the radiant light source for each spectral band is considered based on the target’s global spectral radiant energy ratio. A multi-spectral common aperture optical system will fuse the modulated multi-spectral simulated image, as shown in Figure 3b.

(3) Local radiant grayscale modulation

The multi-spectral local spectral ratio modulation is accomplished by modulating the display device’s grayscale, as shown in Figure 3c, based on the difference between the modulated average global spectral radiant energy ratio and the target local spectral radiant energy ratio.

## 3. Research on Global–Local Multi-Spectral Radiation Features Modulation Algorithm

### 3.1. Design of Algorithm Flow

**Figure 4:**
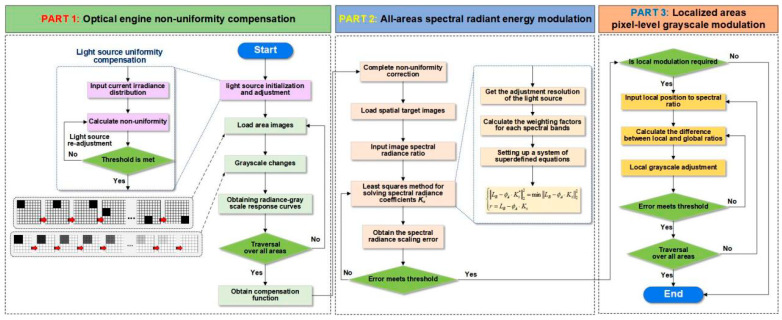
Flowchart of multi-spectral modulation algorithm.

According to the constructed modulation link, the flowchart of the multi-spectral modulation algorithm is shown in Figure 4.

The algorithm consists of three components: optical engine non-uniformity compensation, global spectral radiant energy modulation, and local radiant grayscale modulation.

(1) Part I: Optical engine non-uniformity compensation

Firstly, the radiation sources in the three spectral bands are initialized to ensure the irradiation uniformity of the sources themselves. On this basis, for a certain area using two-point and multi-point calibration methods, the irradiation response function under different grayscale is fitted using least squares, traversing the areas one by one in turn and finally generating the compensation calibration function.

(2) Part II: Global spectral feature modulation

According to the global spectral radiant energy ratio of the target image, the global spectral features are modulated by adjusting the weight factors of the four sub-spectral bands. The least squares method is used to find the minimum value of the residual between the simulated spectral ratios and the target spectrum.

(3) Part III: Local spectral feature modulation

The local spectral feature modulation is finished when the grayscale modulation factor is computed to modulate the grayscale value of the local pixel points of the display devices in each band by comparing the difference between the spectral ratio of the local feature areas in the target image and the overall ratio.

### 3.2. Optical Engine Non-Uniformity Compensation

(1) Light source initialization and adjustment

We select the working spectral band, obtain the maximum value of irradiance $k_{\max}$ and the minimum value of irradiance $k_{\min}$ on the irradiance surface at the outlet, calculate the irradiance non-uniformity ε of the light source according to $\varepsilon =\left|\frac{k_{\max}-k_{\min}}{k_{\max}+k_{\min}}\right|$, and take the non-uniformity not greater than $\varepsilon_{st}$ as a criterion for the light source to be qualified. When the non-uniformity is poor, the light source needs to be adjusted until the non-uniformity meets the threshold (25%).

(2) Load area image

We let the resolution of the display device be m × n, divided into p × q areas on the target surface, and load the image in each area in turn. At this time, we let the theoretical value of the output energy distribution at the image surface be matrix $S_{m\times n}$, and the corresponding energy on each pixel be $S_{i,j}\left(G\right)$. The energy distribution matrix of the radiation surface of the light source caused by the non-uniformity of illumination from the light source is $K_{m\times n}$, and the corresponding irradiance at each pixel position is denoted as $k_{ij}$. The modulation matrix of the display device itself for the effect of the non-uniformity is $G_{m\times n}$, and the corresponding irradiance at each pixel position is denoted as $g_{ij}$. The actual measured output energy at the image surface is $S_{m\times n}\left(G\right)$, which can be expressed as follows:

(1) $$S_{m\times n}\left(G\right)=S_{E}\left(m\times n\right)\Theta K_{m\times n}\Theta G_{m\times n}$$

In Equation (1), $S_{m\times n}\left(G\right)=\left[\begin{array}{ccc} S_{11}\left(G\right) & \cdots & S_{1n}\left(G\right)\\ \vdots & \ddots & \vdots \\ S_{m1}\left(G\right) & \cdots & S_{mn}\left(G\right) \end{array}\right]$, $S_{E}\left(m\times n\right)=\left[\begin{array}{ccc} s_{11} & \cdots & s_{1n}\\ \vdots & \ddots & \vdots \\ s_{m1} & \cdots & s_{mn} \end{array}\right]$, $K_{m\times n}=\left[\begin{array}{ccc} k_{11} & \cdots & k_{1n}\\ \vdots & \ddots & \vdots \\ k_{m1} & \cdots & k_{mn} \end{array}\right]$, $G_{m\times n}=\left[\begin{array}{ccc} g_{11} & \cdots & g_{1n}\\ \vdots & \ddots & \vdots \\ g_{m1} & \cdots & g_{mn} \end{array}\right]$.

(3) Grayscale change

The k bits display device pixel 2^k^ grayscale is subdivided into 2^t^ grayscale intervals (*t* < *k*), and the grayscale is incremented step by step from low to high. At this time, the display device adjacent grayscale values $G_{1}$ and $G_{2}$ are used as a calibration point, and we measure the corresponding response output $S_{i,j}\left(G_{1}\right)$ and $S_{i,j}\left(G_{2}\right)$, respectively, to solve for the resolution at this time for the average of the m×n display device spoke luminance response:

(2) $$\bar{S}\left(G\right)=\frac{1}{m\cdot n}\cdot \sum_{i}^{m}\sum_{j}^{n}{\bar{S}}_{i,j}\left(G\right)$$

In Equation (2), $\bar{S}\left(G\right)$ is the average radiance response of the display device.

(4) Obtain radiance–grayscale response curve

The compensation parameters for the adjacent grayscale intervals are determined using the coordinate points $\left(\bar{S}\left(G_{1}\right),G_{1}\right)$ and $\left(\bar{S}\left(G_{2}\right),G_{2}\right)$ corresponding to the average grayscale of the adjacent grayscale values $G_{1}$ and $G_{2}$ points. From this, it can be deduced that the output irradiance $S_{i,j}\left(G\right)$ and the compensated irradiance ${\hat{S}}_{i,j}\left(G\right)$ of the display device are met between the input of any gray level G:

(3) $$\frac{{\hat{S}}_{i,j}\left(G\right)-\bar{S}\left(G_{1}\right)}{\bar{S}\left(G_{2}\right)-\bar{S}\left(G_{1}\right)}=\frac{S_{i,j}\left(G\right)-S_{i,j}\left(G_{1}\right)}{S_{i,j}\left(G_{2}\right)-S_{i,j}\left(G_{1}\right)}$$

It can be derived that

(4) $$\left\{\begin{array}{l} {\hat{S}}_{i,j}\left(G\right)=\frac{\bar{S}\left(G_{2}\right)-\bar{S}\left(G_{1}\right)}{S_{i,j}\left(G_{2}\right)-S_{i,j}\left(G_{1}\right)}\cdot S_{i,j}\left(G\right)+\bar{S}\left(G_{1}\right)-\frac{\left[\bar{S}\left(G_{2}\right)-\bar{S}\left(G_{1}\right)\right]\cdot S_{i,j}\left(G_{1}\right)}{S_{i,j}\left(G_{2}\right)-S_{i,j}\left(G_{1}\right)}\\ {{\hat{S}}_{i,j}}^{\prime }\left(G\right)=\frac{\bar{S}\left(G_{2}\right)-\bar{S}\left(G_{1}\right)}{S_{i,j}\left(G_{2}\right)-S_{i,j}\left(G_{1}\right)}=k_{i,j}\left(G\right)\\ {\hat{S}}_{i,j}\left(0\right)=\bar{S}\left(G_{1}\right)-\frac{\left[\bar{S}\left(G_{2}\right)-\bar{S}\left(G_{1}\right)\right]\cdot S_{i,j}\left(G_{1}\right)}{S_{i,j}\left(G_{2}\right)-S_{i,j}\left(G_{1}\right)}=b_{i,j}\left(G\right) \end{array}\right.$$

Equation (4) can be simplified to

(5) $${\hat{S}}_{i,j}\left(G\right)=k_{i,j}\left(G\right)G+b_{i,j}\left(G\right)$$

The error fitting response function of the display device in the range of [$G_{1},G_{2}$] grayscale input interval is Equation (5), where $k_{i,j}$ is the slope and $b_{i,j}$ is the intercept.

(5) Complete areas traversal

We determine whether the current compensation of all p × q areas is completed. If there are still areas that have not been traversed, it is necessary to reload the image of the area; all traversed can be skipped to the next step.

(6) Obtain the compensation function

For the pixel point (i, j) on the display device, the DMD input grayscale value is G. The slopes and intercepts of the linearized response curve for this point are $k_{i,j}$ and $b_{i,j}$, the slopes and intercepts of the full-area averaged response curve are ${\bar{k}}_{i,j}$ and ${\bar{b}}_{i,j}$, and the calibrated intermediate grayscale value for this point is

(6) $$G_{2}=\frac{{\bar{k}}_{i,j}G_{1}+{\bar{b}}_{i,j}-b_{i,j}}{k_{i,j}}$$

The grayscale correspondence before and after linearization is queried with the intermediate grayscale value G_2_ to obtain the corrected grayscale value $g_{ij}^{\prime }$ of the point, and, finally, the compensation error matrix can be generated according to the corrected grayscale value as $G_{m\times n}^{\prime }$:

(7) $$G_{m\times n}^{\prime }=\left[\begin{array}{ccc} g_{11}^{\prime } & \cdots & g_{1n}^{\prime }\\ \vdots & \ddots & \vdots \\ g_{m1}^{\prime } & \cdots & g_{mn}^{\prime } \end{array}\right]$$

The $G_{m\times n}^{\prime }$ is the optical engine non-uniformity compensation function, which can be assigned as an initial value to the corresponding pixel point of the display device to compensate for the optical engine non-uniformity until the compensation of all spectral bands is completed. Then, we skip to the next section.

### 3.3. Global Spectral Feature Modulation

(1) Load space target image

The target image is sent to the display device corresponding to each spectral band; the spectral radiance ratio of the image is input and read, and it is input as a true value to the modulation algorithm unit.

(2) Least squares method for solving spectral radiance coefficients

According to Planck’s blackbody radiation law and Wien’s displacement law [22], we obtain

(8) $$\left\{\begin{array}{c} M\left(\lambda ,T\right)=\frac{c_{1}}{\lambda^{5}}\frac{1}{\exp\left(\frac{c_{2}}{\lambda T}\right)-1}\\ \lambda_{m}\cdot T=2898 \end{array}\right.$$

In Equation (8), $c_{1}=3.7418\times 10^{8}$ W·m^-2^·μm^-4^, $c_{2}=14388$ μm·K, T is the absolute temperature at which the blackbody is currently located, λ is wavelength, $M\left(\lambda ,T\right)$ is expressed as the spectral radiance, and $\lambda_{m}$ is the wavelength corresponding to the peak blackbody irradiance at that temperature. From this, a mathematical model of spectral radiant energy modulation can be established as follows:

(9) $$\left\{\begin{array}{l} \phi_{UV-VIS}=k_{1}\int_{\lambda_{1}}^{\lambda_{2}}I\left(\lambda \right)d\lambda +k_{2}\int_{\lambda_{3}}^{\lambda_{4}}I\left(\lambda \right)d\lambda \\ \phi_{MWIR-LWIR}=k_{3}\int_{\lambda_{5}}^{\lambda_{6}}M\left(\lambda +\Delta \lambda_{M},T\right)d\left(\lambda +\Delta \lambda_{M}\right)+k_{4}\int_{\lambda_{7}}^{\lambda_{8}}M\left(\lambda +\Delta \lambda_{L},T\right)d\left(\lambda +\Delta \lambda_{L}\right)\\ \Delta \lambda =-\frac{2898\cdot \xi }{T_{1}\cdot T_{2}}\\ \phi =\phi_{UV}+\phi_{VIS}+\phi_{MWIR}+\phi_{LWIR} \end{array}\right.$$

In Equation (9), k is the radiant power coefficient of the light source, ϕ is the total radiant energy of the target image, ξ is the amount of change in the temperature of the infrared blackbody, and Δλ is the amount of displacement of the peak wavelength due to temperature.

At this point, $\Delta \phi_{UV}$, $\Delta \phi_{VIS}$, $\Delta \phi_{MWIR}$, and $\Delta \phi_{LWIR}$ are each radiation source’s minimum radiation change units. A system of over-determined equations can be constructed concerning the modulation coefficients of the four spectral bands, the smallest radiation unit, and the measured values:

(10) $${\left[\begin{array}{cccccc} k_{1} & k_{1} & k_{1} & 0 & 0 & 0\\ k_{2} & k_{2} & 0 & k_{2} & 0 & 0\\ k_{3} & 0 & 0 & 0 & k_{3} & 0\\ k_{4} & 0 & 0 & 0 & 0 & k_{4} \end{array}\right]}^{T}\left[\begin{array}{l} \Delta \varphi_{UV}\\ \Delta \varphi_{VIS}\\ \Delta \varphi_{UV}\\ \Delta \varphi_{UV} \end{array}\right]={\left[U_{0}^{\prime }\;U_{1}^{\prime }+U_{2}^{\prime }\;U_{1}^{\prime }\;U_{2}^{\prime }\;U_{3}^{\prime }\;U_{4}^{\prime }\right]}^{T}$$

The least squares solution can be used to solve for Equation (10), and Equation (10) can be expressed in the form of $K_{x}\cdot \Delta \phi =U^{\prime }$. Then, we have

(11) $$\left\{\begin{array}{l} {\left\|U^{\prime }-\Delta \phi \cdot K_{x}^{*}\right\|}_{2}^{2}=\min{\left\|U^{\prime }-\Delta \phi \cdot K_{x}\right\|}_{2}^{2}\\ r=U^{\prime }-\Delta \phi \cdot K_{x}^{*} \end{array}\right.$$

In Equation (11) $K_{x}^{*}={\left[k_{1}^{*},k_{2}^{*},k_{3}^{*},k_{4}^{*}\right]}^{T}$, $K_{x}^{*}\in R_{n}$ is the least squares solution of the system of Equation (11) and r is the residual.

(3) Obtain the spectral radiation ratio error

According to the obtained least squares solution $K_{x}^{*}$, we calculate the error between the measured value of global radiant energy $U^{\prime }$ and the theoretical true value ϕ. If the error exceeds the threshold value (5%), we increase the measurement times and reset the least squares solution until the spectral radiant proportionality error meets the requirement.

### 3.4. Local Spectral Feature Modulation

(1) Input local position coordinates and spectral ratio

If feature areas in the target image require local spectral modulation, then we read the pixel position coordinates and local spectral radiation ratio of the areas.

(2) Calculate the error difference between the local and global ratios

We analyze the radiant energy theoretical ratio in the local area and select the highest energy band as the standard band for modulation, and the theoretical radiant energy ratio of the standard band is *S*:

(12) $$S={\left\|S_{UV},S_{VIS},S_{MW},S_{LW}\right\|}_{\max}$$

In the global radiation ratio $\left[S_{UV}^{\prime },S_{VIS}^{\prime },S_{MW}^{\prime },S_{LW}^{\prime }\right]$, the value of the percentage of radiant energy corresponding to the standard band is noted as $S^{\prime }$.

(3) Local grayscale modulation

Based on the global and local ratio of radiant energy, the local pixel grayscale transformation factors $B_{x}$ for each band are obtained:

(13) $$B_{x}=\frac{S^{\prime }}{S\cdot S_{x}^{\prime }}S_{x}$$

In Equation (13), x represents different spectral bands. The grayscale value of a certain spectral segment that needs to be modulated locally can be expressed as

(14) $$g_{ij}^{\prime \prime }=g_{ij}^{\prime }\cdot \left[\frac{S^{\prime }\cdot S_{x}\left(g_{ij}-1\right)}{S\cdot S_{x}^{\prime }}+1\right]$$

In Equation (14), $g_{ij}$, $g_{ij}^{\prime }$, and $g_{ij}^{\prime \prime }$ are the initial grayscale of the (i, j) pixel of the display device, the compensation value of the grayscale non-uniformity, and the grayscale modulation value, respectively.

Finally, the target image that completes the local radiation grayscale modulation is sent to the corresponding display device. At this time, the radiation energy corresponding to the (i, j) pixel at the output image plane of the system is $S_{ij}$, which can be expressed as

(15) $$S_{ij}=\sum_{x=1}^{4}{ℜ}_{x}\cdot {s_{ij}}^{px}\cdot {g_{ij}^{\prime \prime }}^{qx}$$

In Equation (15), ℜ is the transfer efficiency of the system, and p and q are the weights of the optical engine and display device in the transfer function.

(4) Judging whether the ratio error meets the threshold and whether the area traversal is completed

We judge whether the difference between the measured value of radiant energy $U^{\prime \prime }$ of the local area after modulation and the theoretical true value of $\phi^{\prime }$ is qualified; if it exceeds the threshold value (5%), then we return to readjust the grayscale until qualified. We judge again whether to complete the traversal modulation of all feature areas. After completion, the algorithm ends.

## 4. Experimentation and Discussion

### 4.1. Experimental Design

In order to verify the improvement of the proposed global–local multi-spectral spectral feature modulation algorithm compared with the traditional modulation algorithm, three sets of comparative experiments were designed: the optical engine non-uniformity compensation experiment, the global spectral radiant energy modulation experiment, and the local radiant grayscale modulation experiment.

(1) Optical engine non-uniformity compensation experiment

For each spectral band, the 20 × 20 checkerboard method was used to measure the radiant energy value on the irradiated surface, and the radiant energy distribution of the image position before and after the algorithm compensation was obtained.

(2) Global spectral radiant energy modulation experiment

The spatial target image with known spectral radiation characteristics is used as the input signal of the experimental platform, the global spectral modulation algorithm calculates the optimal spectral ratio, and the spectral radiation energy modulation of the UV, VIS, MWIR, and LWIR spectral bands is accomplished. The spectral radiation curves of the full-spectrum bands and sub-spectral bands are measured. The radiation energy ratio error of each spectral band is calculated using the global modulation algorithm.

(3) Local radiant grayscale modulation experiment

Two spatial target images with multiple local spectral radiation characteristics are used as input signals for the experimental platform. The local radiation grayscale modulation algorithm differentiates the grayscale modulation of the display device according to the spectral radiation ratio of the characteristic areas. The spectral radiation ratios of each area are also measured, and the local radiation energy ratio error is calculated.

The names and the purpose of the three groups of experiments are shown in Table 1.

### 4.2. Experimental Platform Construction

**Figure 5:**
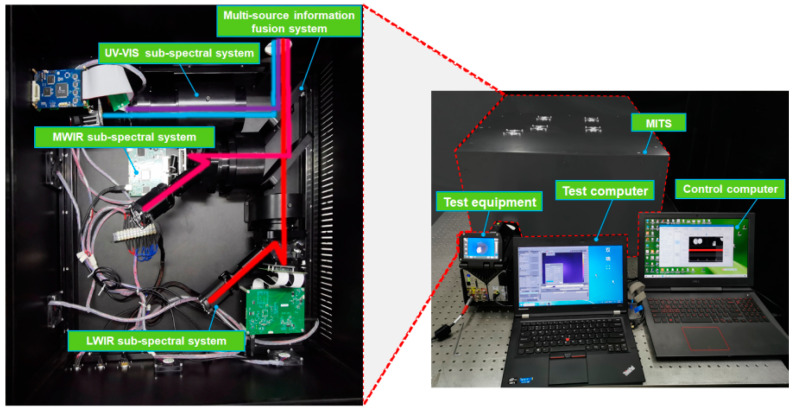
Experimental platform of MITS.

We utilized the UV–VIS sub-spectral system (0.32–0.75 μm), the MWIR sub-spectral system (3–5 μm), the LWIR sub-spectral system (8–12 μm), the multi-source information fusion system, a control computer, a test computer, an FLIR X6530Sc camera (3–5 μm), a Telops FAST V1kx camera (7.5–11.5 μm, USA), an Optosky ATP7820 spectrometer (0.2–13 μm, China), an RD-2000F irradiance meter (0.3–2.5 μm, China), and an RD-2000F irradiance meter (0.3–2.5 μm, China). The IR optical engine resolution is 1024 × 768, the UV–VIS optical engine resolution is 2048 × 1536, the blackbody radiator temperature range is 300–900 K, the UV LED power is 8 W, and the visible–ultraviolet LED power is 8 W. The experimental platform setup is shown in Figure 5.

### 4.3. Experimental Results

#### 4.3.1. Optical Engine Non-Uniformity Compensation Experiment

**Figure 6:**
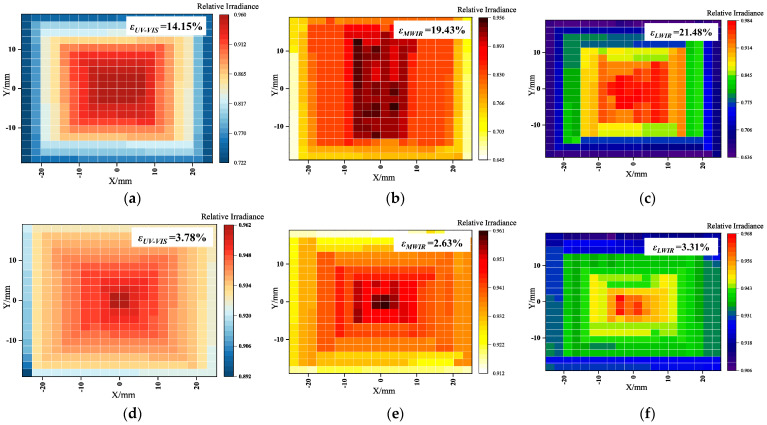
Test results of each spectral band before and after non-uniformity compensation. (**a**–**c**) The results of the non-uniformity tests in the UV–VIS, MWIR, and LWIR bands before compensation. (**d**–**f**) The results of the non-uniformity tests in the three spectral bands after compensation.

Measurements on the irradiance surfaces in the UV–VIS, MWIR, and LWIR spectral bands are shown in Figure 6. The irradiance test results are averaged over several measurements, and the measured radiant energy is finally normalized. The maximum value of the radiant energy $k_{\max}$ and the minimum value of the radiant energy $k_{\max}$ on the outgoing image plane of the optical engine before and after compensation were found, respectively. Substituting them into $\varepsilon =\left|\frac{k_{\max}-k_{\min}}{k_{\max}+k_{\min}}\right|$, the non-uniformity compensation before and after compensation can be calculated as 3.78%, 2.62%, and 3.31%, respectively. Compared with the pre-compensation, they are reduced by 73.32%, 86.53%, and 84.60%, respectively, as shown in Table 2; the non-uniformity after compensation by the algorithm is clearly reduced.

#### 4.3.2. Global Spectral Radiant Energy Modulation Experiment

**Figure 7:**
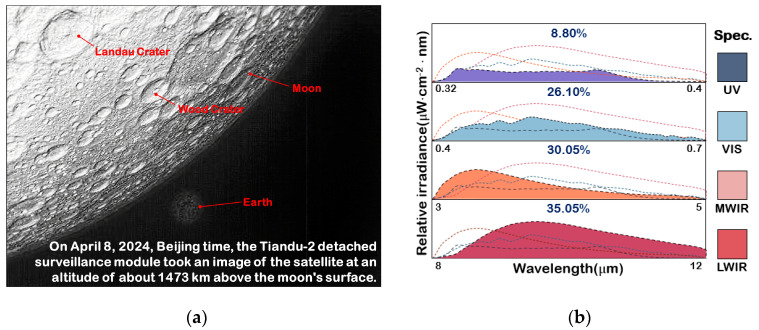
Simulated target images. (**a**) Moon and Earth group image. (**b**) Global spectral radiant energy ratio.

The group photo of the Moon and the Earth [23] (the Moon is in the foreground and the smaller object in the background is the Earth) taken by the multi-source optical payload of China’s Tiandu-2 experimental satellite in lunar orbit is used as the input object of the experiment, as shown in Figure 7a. The ratio of multi-spectral spectral radiation of the image at the current moment can be known from the satellite’s spectral data, as shown in Figure 7b.

**Figure 8:**
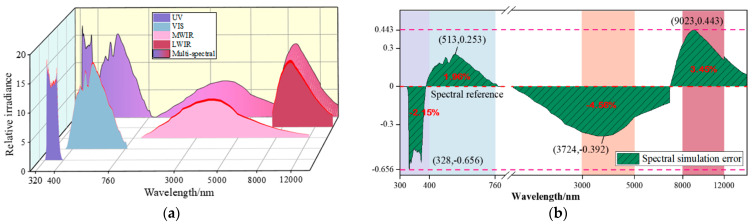
Global spectral radiation energy modulation test results. (**a**) Spectral radiation simulation results. (**b**) Spectral simulation error.

After completing the global modulation, the spectral radiation results of the sub-spectral bands as well as the multi-spectral band fusion are shown in Figure 8a. The ratio simulation error ΔS between multi-spectral bands can be expressed as $\frac{S_{i}-S_{0}}{S_{0}}$, where $S_{i}$ is the actual proportion of total radiant energy accounted for by each sub-spectral band. $S_{0}$ is used as the theoretical true value of the proportion of radiant energy accounted for by each sub-spectral band of the target image. The corresponding spectral simulation error ΔS for each sub-spectral band is shown in Figure 8b.

As can be seen from Figure 8, the energy ratios of the UV, VIS, MWIR, and LWIR spectral bands after global spectral radiant energy modulation are very close to the energy ratios in Figure 7b, and the maximum spectral simulation error is only −4.56%.

#### 4.3.3. Local Radiant Grayscale Modulation Experiment

**Figure 9:**
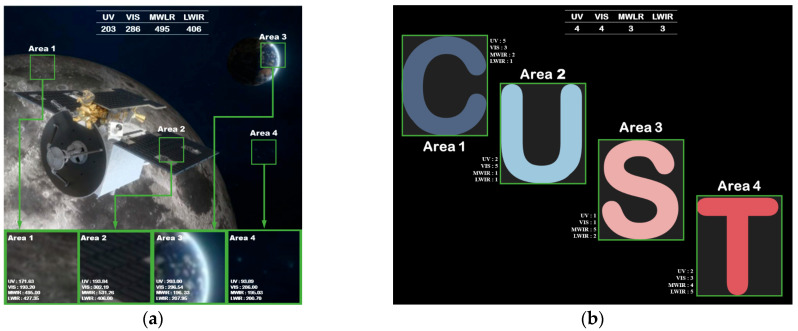
Simulated target images. (**a**) Flight simulation of Tiandu-2. (**b**) Multi-spectral simulation of CUST.

The input images of the radiation grayscale control experiment should be multi-spectral images with more obvious local spectral features. In this paper, the flight simulation image of Tiandu-2 and the simulation image of Changchun University of Science and Technology (CUST) with known spectral scale are used as the input objects of the experiment. In the two images, four feature areas with different spectral radiation ratios are taken, shown in Figure 9a,b, respectively.

**Figure 10:**
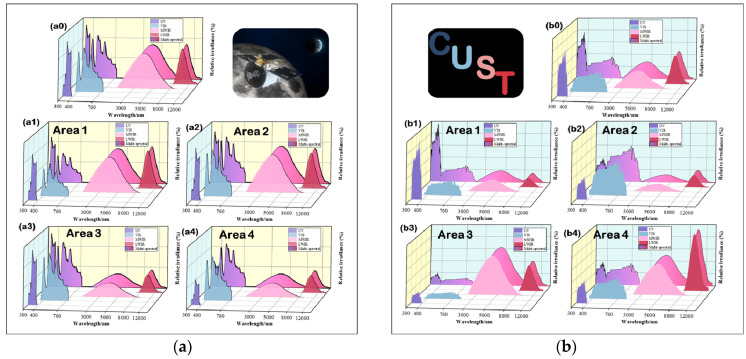
Local radiation grayscale modulation test results. (**a**) Simulation test results of Figure 9a. (**b**) Simulation test results of Figure 9b. (**a0**,**b0**) are the global spectral radiant energy ratio, (**a1**–**a4**,**b1**–**b4**) are the local spectral radiant energy ratio.

According to the global spectral radiant energy ratio data of the image, the spectral ratio is modulated by adjusting the light source output energy and ratio, and the global spectral radiant energy test results of the two images are shown in Figure 10(a0) and Figure 10(b0), respectively. On this basis, the algorithm completes the local radiation grayscale modulation for the spectral radiation energy ratio of the feature areas. The spectral radiant energy test results of the feature areas are shown in Figure 10(a1)–Figure 10(a4) and Figure 10(b1)–Figure 10(b4), respectively.

**Figure 11:**
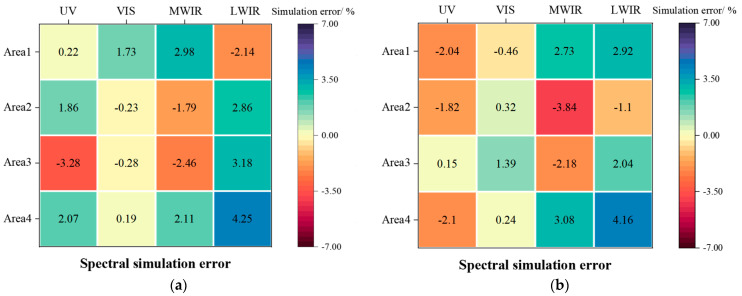
Local spectral simulation errors. (**a**) Simulation error of Figure 10a. (**b**) Simulation error of Figure 10b.

Using $\frac{S_{i}-S_{0}}{S_{0}}$, the spectral simulation errors of the spectral bands of the feature areas in the two images in Figure 11 can be obtained by comparing the test results in Figure 10 with the theoretical truth of the simulation image in Figure 9, respectively. The results are shown in Figure 11, and the maximum spectral simulation error does not exceed 4.25%. It shows that the multi-spectral modulation algorithm can accomplish the arbitrary control of the global–local spectral radiant energy ratio. It also proves that the method of local grayscale modulation of radiant energy has excellent reliability.

### 4.4. Discussion and Comparison

A comparison of the reported multi-spectral target simulation methods with the multi-spectral space target simulation modulation algorithm proposed in this study is given in Table 3.

As Table 3 clearly shows, most of the current studies’ band ranges are narrow or composite near-spectral bands and cannot modulate the spectral radiation ratio quantitatively. A few studies can complete the spectral modulation of visible and near-infrared bands using a broad-spectrum source with a modulation system. However, the simulated objects are limited to static point targets. They cannot modulate the local radiant grayscale, which cannot be used for the performance verification of multi-spectral space cameras.

Compared with related studies, the multi-spectral space target simulation modulation algorithm with combined global–local spectral features proposed in this study is the only multi-spectral target simulation modulation algorithm that can take into account the combined global–local spectral features and the local details of the spectral radiance ratio modulation in the world. At the same time, the working band of this study can cover the UV to LWIR bands, which are at the highest level in the world, and the global simulation accuracy is comparable to the highest level in the world.

## 5. Conclusions

Aiming to address the problem of the lack of local spectral modulation ability in space target simulation, this paper proposes a multi-spectral spectral modulation algorithm for space target simulation with combined global–local spectral features. The overall architecture of the series-parallel MITS, which consists of the UV–VIS sub-spectral system, the MWIR sub-spectral system, the LWIR sub-spectral system, and the multi-source information fusion system, was constructed. A global–local multi-spectral spectral feature modulation link consisting of optical engine non-uniformity compensation for each spectral band, global multi-spectral radiant energy proportional modulation, and local spectral radiant energy proportional modulation was developed.

On this basis, a global–local multi-spectral feature modulation algorithm was proposed, and the algorithm flow and three algorithm modules, including optical engine non-uniformity compensation, global spectral radiant energy modulation, and local radiant grayscale modulation, were designed. Three sets of experiments were designed, the experimental platform was built, and the combined images of the Moon and the Earth captured by the multi-source optical payload of China’s Tiandu-2 experimental satellite in lunar orbit were used to verify the correctness and advancement of the methodology proposed in this study. The experiment results show that the non-uniformity of the optical engine in the three bands was reduced from 14.15–21.48% before the algorithm compensation to 2.62–3.78% after the compensation, which was optimized by 73.32–86.53%. The simulation error of global spectral radiant energy modulation was less than −4.56%. The spectral simulation error for local radiant grayscale modulation was less than 4.25%.

Comparison with other multi-spectral target simulation methods was made, and the results demonstrate that the working band and global simulation accuracy of this study are among the most advanced internationally. This study is the only multi-spectral target simulation modulation algorithm in the world that can take into account the global whole and local details of the spectral radiance proportional modulation.

In the future, we aim to expand multi-spectral subdivisions, refine local spectral feature simulation capabilities, explore the coupling mechanism of geometric and radiometric properties for multi-spectral space targets, ensure architectural stability, and further enhance the theoretical framework of multi-spectral space target simulation and the ground-based semi-physical simulation link for optical loads.

## Figures and Tables

**Table 1:** Experimental design table.

Serial Number	Experiment Name	Purpose
1	Optical engine non-uniformity compensation experiment	Testing the effectiveness of the algorithm in optical engine non-uniformity compensation for each sub-spectral band.
2	Global spectral radiant energy modulation experiment	Testing the modulation accuracy of the algorithm on the global radiant energy ratio of each spectral band.
3	Local radiant grayscale modulation experiment	Testing the accuracy of the algorithm for modulation of localized radiant energy ratios.

**Table 2:** Non-uniformity before and after compensation for each spectral band.

Spectral Band/μm	Non-Uniformity/%	Optimization Ratio/%
0.32–0.76	Before compensation: 14.15	73.32
	After compensation: 3.78	
3–5	Before compensation: 19.43	86.53
	After compensation: 2.62	
8–12	Before compensation: 21.48	84.60
	After compensation: 3.31	

**Table 3:** Comparison of relevant studies.

Related Studies		Spectral Range/μm	Target Type	Simulation Accuracy/%	Global/Local Modulation
In 2013, OPTRA Inc.’s study [11]		3–5 and 8–12	Dynamic surface	/	/
In 2016, Inframet Inc.’s study [12]		1.1–8 and 0.4–12	Static surface	/	Global
In 2016, SBIR Inc.’s study [14]		3–5 and 8–12	Dynamic surface	/	Global
In 2018, Pan’s study [15]		3.7–4.8 and 8–12	Dynamic surface	/	Global
In 2023, Liu’s study [16]		0.45–1	Static point	±4.996%	/
In 2024, Yun’s study [17]		0.4–0.8	Static point	±3.50%	/
This study	Global spectral feature modulation algorithm	0.32–0.76 and 3–5 and 8–12	Dynamic surface	±4.56%	Combined global–local
	Local spectral feature modulation algorithm		Dynamic surface	±4.25%	

## Data Availability

The raw data supporting the conclusions of this article will be made available by the authors upon request.
